# Assessment of Anticholinesterase Activity of *Gelidiella acerosa*: Implications for Its Therapeutic Potential against Alzheimer's Disease

**DOI:** 10.1155/2012/497242

**Published:** 2012-12-06

**Authors:** Arif Nisha Syad, Karutha Pandian Shunmugiah, Pandima Devi Kasi

**Affiliations:** Department of Biotechnology, Alagappa University, Tamil Nadu, Karaikudi 630 003, India

## Abstract

The effect of various solvent extracts of *Gelidiella acerosa* on acetylcholinesterase (AChE) and butyrylcholinesterase (BuChE) activities was investigated. AChE and BuChE inhibitory activities were analyzed by spectrophotometric method. Phytochemical screening of the compounds present in the solvent extracts was done qualitatively. Characterization of the compounds present in the benzene extract of *G. acerosa* was done by GC-MS analysis. The results showed that, at 487.80 **μ**g/mL, benzene extract showed significant (*P* < 0.05) inhibitory activity against both AChE and BuChE with the percentage of inhibition 54.18 ± 5.65 % (IC_50_ = 434.61 ± 26.53 **μ**g/mL) and 78.43 ± 0% (IC_50_ = 163.01 ± 85.35 **μ**g/mL), respectively. The mode of inhibition exhibited by benzene extract against the AChE and BuChE was found to be competitive and uncompetitive type of inhibition, respectively. Preliminary phytochemical analysis coupled with GC-MS illustrates that the benzene extract possesses high amount of terpenoids, which could be the reason for potential cholinesterase inhibitory activity.

## 1. Introduction

 Alzheimer's disease (AD) is the most common neurodegenerative disorder and the prevalent cause of dementia in elderly population [1]. It is clinically characterized by numerous symptoms such as memory and language impairment, cognitive dysfunction, and behavioral disturbances (i.e., depression, agitation, and psychosis), which become progressively more severe [2]. As the aged population grows, the number of individuals worldwide with AD is expected to rise to 34 million in the next three decades, a dramatic increase from 7.3 million today [3]. This is an alarming prospect, particularly in the absence of effective preventive and therapeutic interventions. The most remarkable biochemical change in AD patients is a reduction of acetylcholine (ACh) levels in the hippocampus and cortex of the brain [4]. Therefore, inhibition of acetylcholinesterase (AChE), the enzyme responsible for hydrolysis of ACh at the cholinergic synapse, is currently the most established approach for treating AD [5]. The clinical efficacy of those AChE inhibitors is thought to result from prolonging the half-life of ACh through inhibition of AChE [6]. Currently, five pharmaceutical drugs representing cholinesterase inhibitors (ChEIs), namely, galantamine, rivastigmine, donepezil, and tacrine, are applied clinically. However they can only offer little more than short-term palliative effects, and moreover these inhibitors suffer from pronounced peripheral side effects [7]. Therefore, there is still a great demand in finding new drug candidates for AD treatment.

Marine resources are the richest source of fauna and flora with untapped potentials. The southern coast of India bears a luxuriant growth of seaweed, and these have been used in Asia for more than 2000 years as a subsidiary food, fertilizers, and animal feed [8]. Recently, edible seaweeds have been shown to exert many positive physiological effects, including antiviral, antitumor, anticancer, hepatoprotective, and antiviral activity [9, 10]. Seaweeds are considered as marine renewable sources and medicinal food of 21st century. The proposed seaweed *G. acerosa* is a perennial red algae (*Rhodophyceae*) widely distributed along the south coastal region, that is, Gulf of Mannar throughout the year. It is widely used in the production of superior quality agar for molecular biology studies and also for the treatment of gastrointestinal disorders [11]. S-ACT-1 a sulfono glycolipid of *G. acerosa* has potent sperm motility stimulating activity *in vitro* [12]. S-PC-1 a sphingosine derivative from *G. acerosa* was found to act as non-steroidal antiprogesterone contraceptives [13]. Reports regarding the pharmacological application of *G. acerosa, *especially in the treatment of neurological disorder, are still at its infancy; hence, the objective of our study is to screen for the ChE inhibitory activity of *G. acerosa, *which is commonly available seaweed in southern coast of India. Preliminary screening for antioxidant activity of seaweed has shown that *G. acerosa* extract exhibited excellent antioxidant activity [14]. As the preliminary work was promising, we intended to study the application of this antioxidant seaweed in inhibiting cholinesterase activity so that it could act as safe multipotent drug for treatment of AD.

## 2. Materials and Methods

### 2.1. Sample Collection

Seaweed *G. acerosa* was collected along the South Indian coastal area, Tamil Nadu, and the species were identified according to Oza and Zaidu [15] and Krishnamurthy and Joshi [16] and further confirmed by Dr. M. Ganesan, Scientist, CSMCRI, Mandapam Camp, Tamil Nadu, and the voucher specimen was deposited at Department of Biotechnology, Alagappa University, under the accession number AUDBTGA20100101.

### 2.2. Preparation of Crude Extracts

The seaweeds were washed with alcohol and water and dried under shade. The dried seaweeds were stored in an airtight container, which was stable for at least 12 months. The air dried seaweeds were powdered and successively extracted with different solvents: petroleum ether, hexane, benzene, dichloromethane, chloroform, ethyl acetate, acetone, methanol, and water in Soxhlet apparatus. The extracts were dried under reduced pressure in vacuum dessicator until dryness and the percentage of yield was calculated. The dried extract was dissolved in distilled water containing less than 0.02% of methanol or Tween 20 as solvents and used for further analysis. The extraction procedures were done at temperature less than 40°C to avoid thermal degradation of the compounds. Yield of the extract was calculated as below:

(1) Yield  of  the  extract=Wt.  of  the  beaker  with  extract−Wt.  of  the  empty  beakerWt.  of  the  sample  in  grams ×100.

### 2.3. Chemicals

Electric eel AChE (Type-VI-S, EC 3.1.1.7, Sigma) and horse serum BuChE (EC 3.1.1.8, Sigma) were used as enzyme source, while acetylthiocholine iodide (ATCI) and butyrylthiocholine iodide (BuTCI) (Himedia laboratories, Mumbai, India) were employed as substrates of the reaction. 5,5′-Dithio-bis(2-nitrobenzoic)acid (DTNB) (Himedia laboratories, Mumbai, India) was used as chromogen for the measurement of the cholinesterase activity. All other chemicals used were of highest purity grade and commercially available.

### 2.4. Determination of AChE and BuChE Inhibitory Activities

AChE and BuChE inhibitory activities were measured by slightly modifying the spectrophotometric method developed by Ellman et al. [17] and Ingkaninan et al. [18]. Briefly, 10 *μ*L (0.09 U/mL) of AChE/BuChE solution was incubated with 10–50 *μ*L of various concentrations of *G. acerosa* extract (97.56, 195.12, 292.68, 390.24, 487.80 *μ*g/mL) in 0.05 M Tris-HCl buffer (pH 8.0) for 45 min at RT. After incubation, 125 *μ*L of 3 mM DTNB was added, and the total volume was made up to l mL with Tris-HCl buffer (pH 8.0). Enzyme activity was initiated with the addition of 25 *μ*L of 15 mM ATCI/BuTCI. The hydrolysis of ATCI and BuTCI was monitored by the formation of yellow 5-thio-2-nitrobenzoate anion as a result of the reaction of DTNB with thiocholine, catalyzed by the enzyme at a wavelength of 405 nm utilizing UV-Visible spectrophotometer (U-2800, Hitachi, Japan). Percentage of inhibition of AChE/BuChE was determined by comparison of rates of reaction of samples relative to blank sample (Tris-HCl buffer) using the formula (*E* − *S*)/*E* × 100, where *E* is the activity of enzyme without test sample and *S* is the activity of enzyme with test sample. The experiments were done in triplicates. Donepezil (currently employed anticholinesterase drug) was used as standard.

### 2.5. Phytochemical Analysis

Preliminary phytochemical analysis was carried out on various solvent extracts of *G. acerosa* using standard procedures to identify the constituents as described by Trease and Evans [19], Sofowora [20], and Harborne [21, 22].

#### 2.5.1. Test for Tannins

A few drops of 0.1% ferric chloride were added to the sample and observed for the formation of brownish green or a blue-black coloration.

#### 2.5.2. Test for Flavonoids

About five volumes of dilute ammonia solution were added to a portion of the sample followed by addition of concentrated H_2_SO_4_. A yellow coloration that was observed indicated the presence of flavonoids. The yellow coloration disappeared on standing.

#### 2.5.3. Test for Terpenoids (Salkowski Test)

Five mL of each extract was mixed in 2 mL of chloroform, and concentrated H_2_SO_4_ (3 mL) was carefully added to form a layer. A reddish brown coloration at the interface was formed to show positive results for the presence of terpenoids.

#### 2.5.4. Test for Cardiac Glycosides (Keller-Kiliani Test)

Five mL of each extract was treated with 2 mL of glacial acetic acid containing one drop of ferric chloride solution. This was underlaid with 1 mL of concentrated sulphuric acid. A brown ring at the interface indicates a deoxy sugar characteristic of cardenolides. A violet ring may appear below the brown ring, while in the acetic acid layer, a greenish ring may form just gradually throughout thin layer.

#### 2.5.5. Test for Alkaloids (Dragendorff's Reagent)

1.5 mL of 10% HCl was added to about 5 mL of the extract, and the mixture was heated for 20 min. It was cooled and filtered. 1 mL of Dragendorff's reagent was added. Formation of a reddish or orange colored precipitate indicates the presence of alkaloids [22].

### 2.6. Thin Layer Chromatography (TLC) Identification

Preliminary phytochemical screening was further confirmed by TLC analysis. TLC was performed using Silica gel 60 F254 plates (Merck). For the detection of alkaloids in benzene extract, chloroform/methanol/glacial acetic acid 6 : 1 : 0.1 was used as running solvent, and the plates were detected using Dragendorff's reagent. In the case of terpenoids, separation of benzene extract was performed using petroleum ether/benzene/dichloromethane 3 : 2 : 5 as running solvents. Plates were visualized by spraying with Vanillin-sulphuric acid reagent, heated at 100°C for 10 min, and then evaluated in visible light [23]. Presence of terpenoids was further confirmed using p-anisaldehyde sulphuric acid as spraying agent using petroleum ether/benzene/dichloromethane 2 : 2 : 6 as running solvents. The color spots detected after spraying with reagents were documented.

### 2.7. GC-MS Analysis

The components of benzene extract were analyzed by GC-MS (GC Clarus 500 Perkin Elmer) instrument with capillary column of Elite-5MS [(5% Diphenyl/95% Dimethyl poly siloxane), 30 × 0.25 mm × 0.25 *μ*m df]. The inlet oven temperature was kept at 110°C initially. The injector temperature was kept at 250°C. 2 *μ*L of sample was injected into GC-MS instrument for analysis. For detection, an electron ionization system with ionization energy of 70 eV was used. Helium was used as carrier gas at flow rate of 1 mL/min. The chemical components of the extract were identified by comparing their retention indices (RI) and mass fragmentation patterns with those on the stored NIST library (National Institute of Standards and Technology).

### 2.8. Kinetic Studies

To evaluate the type of inhibition, in which the extract inhibits the enzyme activity, the parameters *K*_*m*_, *V*_max⁡_, and *K*_*i*_ values were determined. AChE and BuChE were incubated with different concentrations of benzene extract of *G. acerosa* with increasing substrate concentration, and the assay was performed as mentioned earlier. All the kinetic parameters were calculated by using the software EZ-Fit model (Perella Scientific Inc, USA).

### 2.9. Calculation of IC_50_

Various concentrations of seaweed extracts were taken for the study, and IC_50_ value (which shows 50% inhibition) was calculated by probit analysis method.

### 2.10. Statistical Analysis

Experimental results concerning this study were represented as mean ± S.D. of three parallel measurements. Analysis of variance was performed by one-way ANOVA. Significant differences between means were determined by Duncan's multiple range tests. *P* values <0.05 were regarded as significant and *P* values <0.01 as very significant.

## 3. Results

### 3.1. Acetylcholinesterase (AChE) and Butyrylcholinesterase (BChE) Inhibitory Activity

The percentage of yield of different solvent extracts of *G. acerosa* is illustrated in Table 1. Various concentrations (97.56–487.80 *μ*g/mL) of different solvent extracts of *G. acerosa* were analyzed for AChE inhibitory activity under *in vitro* condition, and the results are shown in Table 2. Results showed that benzene, ethyl acetate, petroleum ether, chloroform, and acetone showed inhibitory activity against AChE in concentration-dependent manner while hexane, dichloromethane, methanol, and aqueous extract showed no activity against AChE. At 487.80 *μ*g/mL benzene and ethyl acetate extract showed significant (*P* < 0.05) inhibitory activity of 54.18 ± 5.65 and 53.57 ± 9.49% when compared to control, but significantly less when compared to standard drug donepezil whose percentage of inhibition is 98.26 ± 0.236% (IC_50_ value −5.09 ± 0.004 *μ*g/mL). IC_50_ values of benzene and ethyl acetate extract were observed as 434.61 ± 26.53 *μ*g/mL and 444.44 ± 11.63 *μ*g/mL, respectively. Chloroform, acetone, and petroleum ether extract showed moderate inhibitory activity of 37.6 ± 1.17, 45.92 ± 0.679, and 41.36 ± 1.68%, respectively.

 In the case of BuChE, at 487.80 *μ*g/mL benzene, chloroform, ethyl acetate, and petroleum ether showed inhibitory activity when compared to control. Hexane, dichloromethane, methanol, and water extracts showed no inhibitory activity against BuChE (Table 3). Of all the extracts benzene and chloroform extract showed significantly (*P* < 0.05) higher inhibition of 78.43 ± 0% and 56.78 ± 2.13% when compared to control with IC_50_ values of 163.01 ± 85.35 *μ*g/mL and 375 ± 17.170 *μ*g/mL, respectively. Donepezil, a positive control, showed, significantly higher inhibition of 91.57 ± 2.41% (IC_50_ −7.266 ± 0.0065 *μ*g/mL) when compared to seaweed extract. Petroleum ether and ethyl acetate extract exhibited moderate inhibitory activity of 32.85 ± 4.76 and 37.74 ± 4.19%, respectively. From the above results it is clear that of the entire extracts studied, benzene extract inhibited both AChE and BuChE to greater extent while ethyl acetate extract showed moderate activity against both the ChEs (dual cholinergic effect).

### 3.2. Phytochemical Screening

Preliminary phytochemical analysis of different solvent extracts of *G. acerosa *was performed, and the results are tabulated in Table 4. Benzene and ethyl acetate extract showed positive results for the presence of terpenoids, cardiac glycosides, alkaloids, and tannins. Negative results were observed for the presence of steroids, flavonoids, and anthraquinones.

### 3.3. Identification of Chemical Constituents of the Benzene Extract

**Figure 1:**
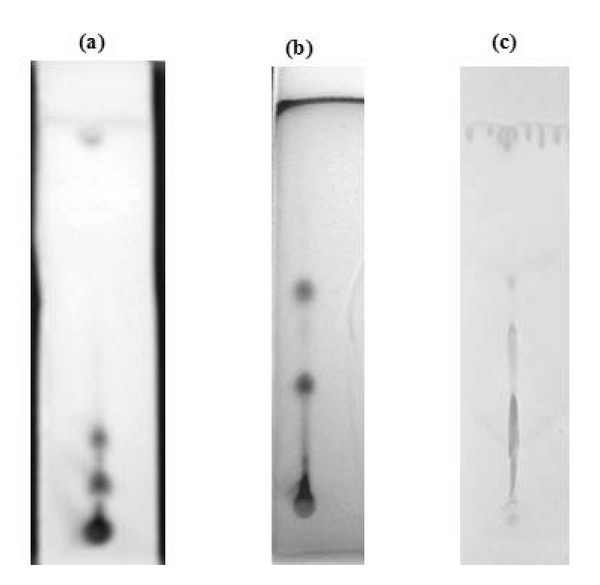
TLC chromatogram of benzene extract showing plates sprayed with (a) p-anisaldehyde sulphuric acid, (b) vanillin-sulphuric acid reagent, and (c) Dragendorff's reagent.

Since the benzene extract showed the highest ChE (AChE and BuChE) inhibitory activity, the chemical constituents were further analyzed by TLC and GC-MS for identifying the compounds responsible for the activity exhibited. In classical TLC analysis, spots with chromatographic behavior identical to terpenoids (purple color when exposed to vanillin-sulphuric acid and violet color when sprayed with p-anisaldehyde reagent) were observed for benzene extract (Figure 1). Orange red colored spots were seen in plates treated with Dragendorff's reagent, which is a positive test for alkaloids (Figure 1) [23, 24]. TLC findings were in agreement with the data of qualitative chemical tests, and the spots characteristic for terpenoids and alkaloids were observed in benzene extract.

A high-resolution mass spectrum equipped with a data system in combination with gas chromatography was used for chemical analysis of benzene extract of *G. acerosa*. A total of 24 compounds were identified from the benzene extract (Table 5). The extract contained a complex mixture consisting of monoterpene and sesquiterpene hydrocarbons. The major compounds detected were 5-Amino-2-methoxyphenol; Eicosane; 2-Pentadecanone,6,10,14-trimethyl-; 1,2-Benzenedicarboxylic acid, diisooctyl ester; 9-Octadecenoic acid, 12-(acetyloxy)-, methyl ester, [R-(Z)]-; Phytol and Lanosta-7,9(11)-diene-3a,18,20-triol. The anticholinesterase activity observed in the benzene extract might be attributed to the presence of the above-mentioned compounds.

### 3.4. Computation of Kinetic Parameters

Kinetic studies were performed using IC_50_ and IC_25_ of benzene extract of *G. acerosa,* and the results were tabulated in Table 6. The results suggest that both AChE and BuChE showed maximum enzyme velocity at the concentration of 15 mM. *K*_*m*_, *V*_max⁡_ and *K*_*i*_ values were determined by plotting Lineweaver-Burk plot. In the case of AChE, an increase in the *K*_*m*_ value (1.729 ± 0.56 nM) was observed without showing any significant change in the *V*_max⁡_ (0.005 ± 0.0002 nmoles/min/mg of protein) when compared to the control (*K*_*m*_ −0.8774 ± 0.41 nM, *V*_max⁡_ −0.0047 ± 0.0002 nmoles/min/mg of protein), which suggests that the type of inhibition involved might be of competitive type [25]. The *K*_*i*_ value obtained for the extract was 202.18 ± 72.20 *μ*g/mL. In the case of BuChE, a decrease in the *K*_*m*_ value (1.086 ± 0.60 nM) was observed when compared to control (3.12 ± 0.87 nM). A slight decrease in the *V*_max⁡_ value (0.0024 ± 0.0001 nmoles/min/mg of protein) was also observed when compared to control (0.0027 ± 0.0002 nmoles/min/mg of protein). The *K*_*i*_ value obtained for the extract was 162.94 ± 71.52 *μ*g/mL. Therefore the results suggest that the type of inhibition involved might be uncompetitive [26].

## 4. Discussion

AD is a progressive neurodegenerative disorder characterized by deficit in cholinergic neurotransmission in basal forebrain. ChEIs represent the most promising therapeutic agents for AD type dementia patients, as shown by the clinical studies on the effects of these drugs on cognition (memory and concentration) and behavioral symptoms (apathy and motor agitation) [27]. Many investigations have previously attempted to develop ChEIs for the treatment of AD, either synthetically or via the exploitation of plants and fungi used in traditional medicine, but research into an effective agent from marine algae is still in its infancy. Preliminary screening studies revealed that only crude methanol extract of *G. acerosa* exhibited antioxidant activity among the various seaweed types used for evaluation [14]. In the present study, we extended this observation to further investigate ChE inhibitory activity using different solvent extracts of *G. acerosa* in cell-free *in vitro* assays.

The main finding of the present study was that benzene extract of *G. acerosa * showed significant dual cholinergic activity; that is, it is active against both AChE and BuChE. Plant extracts, which have dual anti-ChE activity, may be appropriate to patients in moderate stage of AD [27]. Moreover, Hodges [28] demonstrated that inhibition of AChE plays a key role not only in enhancing the cholinergic neurotransmission in the brain but also in reducing the aggregation of *β*-amyloid the key factor in AD. Donepezil the currently used ChEI has been reported to activate *α*-secretase and promote non-amyloidogenic pathway [29]. A recent report also demonstrated that selective BuChE inhibitors reduced amyloid precursor protein processing and A*β* level *in vivo* and *in vitro* [30]. These data suggest that the effects may arise from the interaction of these drugs with amyloid cascade, influencing the expression and metabolic processing of APP and slowing down the major pathological consequences of aggregation [31]. Therefore ChEIs not only increase the level of ACh but also prevent the formation of *β*-amyloidal plaques thereby protecting the neurons from neurodegeneration.

In terrestrial plants the majority of ChEIs were identified to be alkaloids and terpenoids [5, 32] whereas reports regarding ChEI activity of seaweeds are still in its infancy. TLC analysis of benzene extract showed the presence of alkaloids and terpenoids. GC-MS analysis also showed that benzene extract of *G. acerosa* possesses considerable amount of different types of terpenoids. Terpenoids are secondary metabolites synthesized by seaweeds and represent a form of essential oils and are classified according to their isoprene unit such as mono-, sesqui-, di-, and triterpene [33]. They possess higher therapeutic potentials like anticancer, antioxidant, and anti-inflammatory activities either independently or synergistically [34]. Recent findings reveal that terpenoids have potential neuroprotective effects against ischemic and glutamatergic neurotoxicity, 6-hydroxydopamine toxicity, and oxidative stress [33]. Studies on ethanolic extract of *Salvia potentillifolia* show that the essential oils containing mono- and sesquiterpenoids obtained from them exhibit excellent cholinesterase inhibitory activity in *in vitro* condition [35]. Shiomi [36] demonstrated the anticholinesterase activity of monoterpenes isolated from fungi. These terpenoids, on the other hand, due to their small molecular size and lipophilicity, readily cross the blood-brain barrier and are effective in the treatment of AD [37]. Hence the observed cholinesterase inhibitory activity of *G. acerosa* extract might be attributed to the presence of the different types of terpenoids.

## 5. Conclusion

The results obtained from this study clearly indicate that benzene extract of *G. acerosa* has a powerful ChE inhibitory activity under *in vitro* condition. The most probable reason for their potential ChEI activity might be related to the presence of terpenoids. The significance of natural ChEIs from *G. acerosa* will be further characterized, and they will be evaluated for their bioavailability and potential toxicity *in vivo*.

## Figures and Tables

**Table 1:** Percentage of yields (W/W) of different solvent extracts of *Gelidiella acerosa. *

S. No	Solvent extracts	Yield of extract
1	Petroleum ether	0.06%
2	Hexane	0.06%
3	Benzene	0.06%
4	Dichloromethane	0.24%
5	Chloroform	0.06%
6	Ethyl acetate	0.03%
7	Acetone	0.41%
8	Methanol	4.99%
9	Water	5.01%

**Table 2:** Acetylcholinesterase inhibitory activity of different solvent extracts of *Gelidiella acerosa. *

S. no.	Solvent extract	% of inhibition ± S.D^a^					IC_50_ (*μ*g/mL)
		97.56 *μ*g/mL	195.12 *μ*g/mL	292.68 *μ*g/mL	390.24 *μ*g/mL	487.80 *μ*g/mL
1	Donepezil	98.05 ± 0.23	98.05 ± 0.12	98.05 ± 0.30	98.39 ± 0.34	98.26 ± 0.23**	5.0985 ± 0.0046
2	Petroleum ether	9.95 ± 0.65	21.93 ± 2.49	29.20 ± 1.42	36.31 ± 1.18	41.36 ± 1.68	Nil
3	Hexane	NI^b^	NI^b^	NI^b^	NI^b^	NI^b^	Nil
4	Benzene	14 ± 1.5	40 ± 3.05	40 ± 8.08	46 ± 3.46	54.18 ± 5.65**	434.61 ± 26.53
5	Dichloromethane	NI^b^	NI^b^	NI^b^	1.99 ± 0.05	4.34 ± 0.29	Nil
6	Chloroform	26.28 ± 0.95	30.21 ± 0.36	33.44 ± 0.81	35.51 ± 0.67	37.60 ± 1.17	Nil
7	Ethyl acetate	39.28 ± 2.02	42.85 ± 0.68	46.42 ± 0.68	50 ± 0.68	53.57 ± 9.49*	444.44 ± 11.63
8	Acetone	0.19 ± 0. 33	15.88 ± 1.45	31.51 ± 2.07	36.84 ± 0.21	45.92 ± 0.67	Nil
9	Methanol	9.8 ± 0.84	9.95 ± 0.34	10.02 ± 0.55	12.37 ± 3.40	14.82 ± 1.70	Nil
10	Water	NI^b^	NI^b^	NI^b^	NI^b^	6 ± 0.001	Nil

^a^Results were expressed as mean ± SD (*n* = 3).

^b^NI: no inhibition.

*P < 0.05.

**P < 0.01.

**Table 3:** Butyrylcholinesterase inhibitory activities of different solvent extracts of *Gelidiella acerosa. *

S. no.	Solvent extract	% of inhibition ± S.D^a^					IC_50_ (*μ*g/mL)
		97.56 *μ*g/mL	195.12 *μ*g/mL	292.68 *μ*g/mL	390.24 *μ*g/mL	487.80 *μ*g/mL
1	Donepezil	67.71 ± 1.09	85.61 ± 8.50	85.96 ± 1.84	96.65 ± 2.64	91.57 ± 2.41**	7.266 ± 0.0065
2	Petroleum ether	22.17 ± 3.96	24.46 ± 2.28	25.22 ± 4.76	30.57 ± 1.32	32.85 ± 4.76	Nil
3	Hexane	NI^b^	NI^b^	NI^b^	NI^b^	NI^b^	Nil
4	Benzene	43.13 ± 7.67	54.9 ± 9.31	56.86 ± 10.19	70.58 ± 4.76	78.43 ± 0.00**	163.01 ± 85.35
5	Dichloromethane	NI^b^	NI^b^	NI^b^	2.91 ± 0	4.49 ± 1.36	Nil
6	Chloroform	24.68 ± 8.5	39.28 ± 0	45.14 ± 2.71	47.56 ± 0.99	56.78 ± 2.13**	375 ± 17.170
7	Ethyl acetate	15.332 ± 2.03	23.06 ± 4.75	30.39 ± 5.71	32.24 ± 1.58	37.74 ± 4.19	Nil
8	Acetone	12.87 ± 2.5	13.61 ± 2.38	19.51 ± 1.27	23.21 ± 3.38	29.11 ± 3.83	Nil
9	Methanol	NI^b^	NI^b^	1.79 ± 1.27	2.53 ± 0.004	16.44 ± 3.43	Nil
10	Water	NI^b^	NI^b^	NI^b^	NI^b^	NI^b^	Nil

^a^Results were expressed as mean ± SD (*n* = 3).

^b^NI: no inhibition.

*P < 0.05.

**P < 0.01.

**Table 4:** Preliminary phytochemical screening of different solvent extracts of *Gelidiella acerosa. *

S. no.	Compounds	Petroleum ether	Hexane	Benzene	Dichloromethane	Chloroform	Ethyl acetate	Acetone	Methanol	Water	DMSO
1	Alkaloids	++	++	++	++	++	++	−	−	−	−
2	Terpenoids	+	−	++	+	−	+	++	−	−	−
3	Ketonic terpenoids	−	−	+	−	++	−	++	−	−	−
4	Cardiac glycosides	++	+	++	++	++	++	+	+	−	−
5	Tannins	+	+	+	+	+	+	++	++	++	++
6	Flavonoids	−	−	−	−	−	−	−	−	−	−
7	Steroids	−	−	−	−	−	−	−	−	−	−
8	Anthraquinones	−	−	−	−	−	−	−	−	−	−

−: No response; +: low content; ++: high content.

**Table 5:** GC-MS profile of benzene extract of *Gelidiella acerosa. *

S. no.	Retention time	Name of the compound	Molecular formula	Molecular weight
1	4.70	5-Amino-2-methoxyphenol	C_7_H_9_NO_2_	139
2	8.46	2(4H)-Benzofuranone, 5,6,7,7a-tetrahydro-4,4,7a-trimethyl-	C_11_H_16_O_2_	180
3	8.82	2(1H)-Pyridinethione, 3-hydroxy-	C_5_H_5_NOS	127
4	10.17	Eicosane	C_20_H_42_	282
5	11.72	2-Pentadecanone,6,10,14-trimethyl-	C_18_H_36_O	268
6	12.55	Lanosta-7,9(11)-diene-3a,18,20-triol	C_30_H_50_O_3_	458.71
7	12.67	9-Octadecenoic acid, 12-(acetyloxy)-, methyl ester, [R-(Z)]-	C_21_H_38_O_4_	354
8	14.87	Cyclopentanol, 2,4,4-trimethyl-	C_8_H_16_O	128
9	15.00	Phytol	C_20_H_40_O	296
10	15.21	Undecanoic acid, 2-methyl-	C_12_H_24_O_2_	200
11	16.01	2-Piperidinone, N-[4-bromo-n-butyl]-	C_9_H_16_BrNO	233
12	19.29	2,4-Nonadienal	C_9_H_14_O	138.21
13	20.90	1,2-Benzenedicarboxylic acid, diisooctyl ester	C_27_H_46_O	386
14	21.22	n-Decanoic acid	C_10_H_20_O_2_	172.26
15	23.16	Heptanal	C_7_H_14_O	114.19
16	23.77	Acetophenone	C_8_H_8_O	120.15
17	24.86	Benzeneacetic acid, alpha hydroxy-alpha methyl	C_9_H_10_O_3_	166.17
18	26.61	Hexanal	C_6_H_12_O	100.16
19	26.71	Octane	C_8_H_18_	114.23
20	27.19	Dodecane, 1,2-dibromo	C_12_H_24_Br_2_	328.13
21	27.66	Benzoic acid, 4-[(trimethylsilyl)oxy]-, trimethylsilyl ester	C_13_H_22_O_3_Si_2_	282.48298
22	29.32	n-Hexadecanoic acid	C_16_H_32_O_2_	256.4241
23	29.46	3-Undecene, 3-methyl-	C_12_H_24_	168.323
24	30.02	Nonanal	C_9_H_18_O	142.24

**Table 6:** Evaluation of kinetic parameters for AChE and BuChE inhibitory activity of benzene extract of *G. acerosa. *

	AChE		BuChE	
Parameters	Control	Treated	Control	Treated
*K*_*m*_ (nM)	0.8774 ± 0.41	1.729 ± 0.56	3.12 ± 0.87	1.086 ± 0.60
*V*_max⁡_ (nmoles/min/mg of protein)	0.0047 ± 0.0002	0.005 ± 0.0002	0.0027 ± 0.0002	0.0024 ± 0.0001
*K*_*i*_ (*μ*g/mL)	202.18 ± 72.20		162.94 ± 71.52	

## References

[1] Cummings JL, Vinters HV, Cole GM, Khachaturian ZS (1998). Alzheimer’s disease: etiologies, pathophysiology, cognitive reserve, and treatment opportunities. *Neurology*.

[2] Terry RD, Katzman R (1983). Senile dementia of the Alzheimer type. *Annals of Neurology*.

[3] Irizarry M, Hyman B, Batchlor T, Cudkowicz M (2001). Alzheimer’s disease. *Principles of Neuroepidemiology*.

[4] Jaén JC, Gregor VE, Lee C, Davis R, Emmerling M (1996). Acetylcholinesterase inhibition by fused dihydroquinazoline compounds. *Bioorganic and Medicinal Chemistry Letters*.

[5] Schneider LS (1996). New therapeutic approaches to Alzheimer’s disease. *Journal of Clinical Psychiatry*.

[6] Darvesh S, Walsh R, Kumar R (2003). Inhibition of human cholinesterases by drugs used to treat Alzheimer disease. *Alzheimer Disease and Associated Disorders*.

[7] Zarotsky V, Sramek JJ, Cutler NR (2003). Galantamine hydrobromide: an agent for Alzheimer’s disease. *American Journal of Health-System Pharmacy*.

[8] McHugh DJ (2003). A guide to seaweed industry. *FAO Fisheries Technical Paper no.*.

[9] Hayashi K, Mori J, Saito H, Hayashi T (2006). Antiviral targets of a chromene derivative from *Sargassum micracanthum* in the replication of human cytomegalovirus. *Biological and Pharmaceutical Bulletin*.

[10] Dias PF, Siqueira JM, Vendruscolo LF (2005). Antiangiogenic and antitumoral properties of a polysaccharide isolated from the seaweed *Sargassum stenophyllum*. *Cancer Chemotherapy and Pharmacology*.

[11] Prasad K, Siddhanta AK, Ganesan M, Ramavat BK, Jha B, Ghosh PK (2007). Agars of Gelidiella acerosa of west and southeast coasts of India. *Bioresource Technology*.

[12] Premakumara GAS, Ratnasooriya WD, Tillekeratne LMV, Amarasekare AS, Rahman AU (2001). Human sperm motility stimulating activity of a sulfono glycolipid isolated from Sri Lankan marine red alga *Gelidiella acerosa*. *Asian Journal of Andrology*.

[13] Premakumara GAS, Ratnasooriya WD, Tillekeratne LMV (1996). Isolation of a non-steroidal contragestative agent from Sri Lankan marine red alga, *Gelidiella acerosa*. *Contraception*.

[14] Devi KP, Suganthy N, Kesika P, Pandian SK (2008). Bioprotective properties of seaweeds: *in vitro* evaluation of antioxidant activity and antimicrobial activity against food borne bacteria in relation to polyphenolic content. *BMC Complementary and Alternative Medicine*.

[15] Oza RM, Zaidu A (2003). *Revised Checklist of Indian Marine Alage*.

[16] Krishnamurthy V, Joshi HY (1970). *A Check List of Indian Marine Algae*.

[17] Ellman GL, Courtney KD, Andres V, Featherstone RM (1961). A new and rapid colorimetric determination of acetylcholinesterase activity. *Biochemical Pharmacology*.

[18] Ingkaninan K, De Best CM, Van Der Heijden R (2000). High-performance liquid chromatography with on-line coupled UV, mass spectrometric and biochemical detection for identification of acetylcholinesterase inhibitors from natural products. *Journal of Chromatography A*.

[19] Trease GE, Evans WC (1989). *Pharmacognosy*.

[20] Sofowora A (1993). *Medicinal Plants and Traditional Medicine in Africa*.

[21] Harborne JB (1973). *Phytochemical Methods*.

[22] Harborne JB (1980). *Phytochemical Methods- A Guide to Modern Techniques of Plant Analysis*.

[23] Wagner H, Bladt S, Zygainski EM (1984). *Plant Drug Analysis- A Thin Layer Chromatography Atlas*.

[24] Karmarkar SH, Keshavachandran R, Augustin A (2001). Biochemical evaluation of root tubers and in vitro induced callus of adapathiyan (HOLOSTEMMA ADA-KODIEN K. SCHUM.). *Journal of Tropical Agriculture*.

[25] Benie T, Thieulant ML (2003). Interaction of some traditional plant extracts with uterine oestrogen or progestin receptors. *Phytotherapy Research*.

[26] Balzarini J, Hernández AI, Roche P (2003). Non-nucleoside inhibitors of mitochondrial thymidine kinase (TK-2) differentially inhibit the closely related herpes simplex virus type 1 TK and *Drosophila melanogaster* multifunctional deoxynucleoside kinase. *Molecular Pharmacology*.

[27] Giacobini E, Buccafusco JJ (2004). Drugs that target cholinesterase. *Cognitive Enhancing Drugs*.

[28] Hodges JR (2006). Alzheimer’s centennial legacy: origins, landmarks and the current status of knowledge concerning cognitive aspects. *Brain*.

[29] Zimmermann M, Gardoni F, Marcello E (2004). Acetylcholinesterase inhibitors increase ADAM10 activity by promoting its trafficking in neuroblastoma cell lines. *Journal of Neurochemistry*.

[30] Greig NH, Utsuki T, Yu QS (2001). A new therapeutic target in Alzheimer’s disease treatment: attention to butyryloholinesterase. *Current Medical Research and Opinion*.

[31] Mukherjee PK, Kumar V, Mal M, Houghton PJ (2007). Acetylcholinesterase inhibitors from plants. *Phytomedicine*.

[32] Menichini F, Tundis R, Loizzo MR (2009). Acetylcholinesterase and butyrylcholinesterase inhibition of ethanolic extract and monoterpenes from *Pimpinella anisoides* V Brig. (Apiaceae). *Fitoterapia*.

[33] Chang HJ, Kim HJ, Chun HS (2007). Quantitative structure-activity relationship (QSAR) for neuroprotective activity of terpenoids. *Life Sciences*.

[34] Mu L, Kou J, Zhu D, Yu B (2007). Comparison of neuroprotective effects of flavonoids, terpenoids, and their combinations from *Ginkgo biloba* on ischemia-reperfusion—injured mice. *Pharmaceutical Biology*.

[35] Kivrak I, Duru ME, Öztürk M, Mercan N, Harmandar M, Topçu G (2009). Antioxidant, anticholinesterase and antimicrobial constituents from the essential oil and ethanol extract of *Salvia potentillifolia*. *Food Chemistry*.

[36] Shiomi K (1999). Meroterpenoids with various biological activities produced by fungi. *Pure and Applied Chemistry*.

[37] Savelev SU, Okello EJ, Perry EK (2004). Butyryl- and acetylcholinesterase inhibitory activities in essential oils of Salvia species and their constituents. *Phytotherapy Research*.

